# 3D Digital Anatomical Models Based on Computed Tomographic Morphometric Analysis of C1 and C2 for Surgical Navigation

**DOI:** 10.3390/jcm14010243

**Published:** 2025-01-03

**Authors:** Wongthawat Liawrungrueang, Watcharaporn Cholamjiak, Peem Sarasombath

**Affiliations:** 1 Department of Orthopaedics, School of Medicine, University of Phayao, Phayao 56000, Thailand; 2 Department of Mathematics, School of Science, University of Phayao, Phayao 56000, Thailand; watcharaporn.ch@up.ac.th; 3 Department of Orthopaedics, Phramongkutklao Hospital and College of Medicine, Bangkok 10400, Thailand; peems13063@gmail.com

**Keywords:** Atlas (C1), Axis (C2), cervical spine, 3D anatomical models, morphometric analysis, CT scan, surgical navigation

## Abstract

**Background/Objectives:** Injuries involving the Atlas (C1) and Axis (C2) vertebrae of the cervical spine present significant clinical challenges due to their complex anatomy and potential for severe neurological impairment. Traditional imaging methods often lack the detailed visualization required for precise surgical planning. This study aimed to develop high-resolution 3D models of the C1 and C2 vertebrae to perform a comprehensive morphometric analysis, identify gender differences, and assess bilateral symmetry to enhance surgical accuracy. **Methods:** A retrospective analysis was conducted using CT scans from 500 patients aged 18 and older from a single-center hospital. Three-dimensional models were generated using InVesalius 3.1 and visualized with Meshmixer. Morphometric measurements included screw placement angles, lamina length and height, bicortical diameters, and pedicle widths. Statistical analyses were conducted using SPSS, with the Student’s *t*-test applied for gender and bilateral comparisons. **Results:** Significant gender differences were found in certain measurements, such as pedicle width (4.85 ± 0.90 mm in males vs. 4.60 ± 0.85 mm in females, *p* = 0.048) and C2 lamina height (12.90 ± 1.40 mm in males vs. 12.40 ± 1.25 mm in females, *p* = 0.033). Most measurements exhibited bilateral symmetry, supporting their applicability across genders. These results align with previous studies and highlight the importance of tailored surgical approaches. **Conclusions:** Three-dimensional models of the C1 and C2 provide comprehensive morphometric data that enhance preoperative planning and surgical precision. Integrating these models into clinical practice can reduce intraoperative risks and improve patient outcomes in cervical spine surgeries.

## 1. Introduction

The cervical spine plays a vital role in supporting the head, enabling a wide range of motion and protecting critical neurovascular structures. Among its components, the Atlas (C1) and Axis (C2) vertebrae hold unique anatomical and functional significance. C1, a ring-shaped vertebra lacking a body and spinous process, articulates with the occipital condyles to facilitate flexion and extension of the head [1,2,3]. The cervical spine is composed of seven vertebrae, with C1 and C2 classified as “atypical” vertebrae due to their unique anatomical features that support and facilitate the movement of the skull. The C1, a ring-shaped vertebra lacking body and spinous process, articulates with the occipital condyles of the skull to form the occipital-atlanto joint. This joint primarily enables flexion and extension of the head, while also bearing its weight. The C2 is distinguished by its odontoid process (dens), a bony projection that articulates with the anterior arch of the C1 at the median atlantoaxial joint. This configuration allows significant rotation of the head without involving the trunk. Together, the C1 and C2 form the craniovertebral junction, the most flexible segment of the spine, enabling complex head movements. However, their specialized structure and proximity to vital neurological structures make them vulnerable to injuries or pathologies that can result in severe neurological impairments or even mortality [4,5]. Consequently, precise anatomical knowledge is essential for the diagnosis and surgical management of conditions affecting the upper cervical spine [2,3].

Current imaging techniques, including radiographs and computerized tomography scans (CT scans), play a fundamental role in the initial assessment and diagnosis of cervical spine conditions. The advent of three-dimensional (3D) digital anatomical models, derived from high-resolution CT imaging, has revolutionized preoperative evaluation by offering the enhanced visualization of complex anatomical relationships [6]. These models provide surgeons with an invaluable tool for comprehending the spatial orientation of structures, thus facilitating improved surgical precision, particularly in procedures necessitating meticulous screw placement and laminar reconstructions [7,8,9,10].

Despite advancements in imaging and modeling, comprehensive morphometric analyses focusing on gender-based variations and bilateral symmetry within the C1 and C2 vertebrae remain underexplored. Understanding these variations is crucial for developing personalized surgical approaches that can mitigate intraoperative risks and optimize patient outcomes. Accurate anatomical knowledge of C1 and C2 is crucial for managing cervical spine conditions, particularly for surgical interventions requiring precise screw placements and stabilization. Imaging techniques, such as radiographs and CT scans, are indispensable for assessing the structural integrity and morphometry of these vertebrae. Detailed morphometric analysis derived from CT data enables surgeons to plan screw trajectories and avoid complications, such as cortical breaches or neurovascular injuries. Understanding morphometric variations, including those related to gender and symmetry, is critical for tailoring surgical approaches to individual patients. Previous studies have explored the anatomy of C1 and C2. However, there remains a limited comprehensive morphometric analysis that accounts for gender differences and bilateral symmetry. Gender-based anatomical variations, such as differences in pedicle width and lamina dimensions, have been shown to influence surgical outcomes and instrumentation choices. Similarly, evaluating bilateral symmetry can simplify preoperative planning by offering consistent anatomical references. Despite the clinical importance of these factors, there is a gap in the literature addressing their relevance in a detailed and systematic manner [3,7,8].

This study aims to provide a comprehensive morphometric analysis of the C1 and C2 vertebrae, focusing on gender differences and bilateral symmetry. By analyzing data from a large sample of patients, this research seeks to furnish surgeons with detailed anatomical insights that can enhance surgical precision, reduce intraoperative risks, and improve patient outcomes in cervical spine surgeries.

## 2. Materials and Methods

### 2.1. Study Design and Population

This retrospective cross-sectional study was conducted in accordance with the Declaration of Helsinki and received approval from the Institutional Review Board of Phayao University Hospital (IRB Number: HREC-UP-HSST 1.1/009/67) on 20 November 2023. The study analyzed cervical spine CT scans from 500 adult patients aged 18 years or older, drawn from the hospital database. Inclusion criteria required participants to have no history of prior cervical spine surgery, significant spinal deformities, or other conditions that could impact morphometric analysis. CT scans of insufficient quality, such as those with poor resolution or imaging artifacts, were excluded to maintain the integrity of 3D reconstructions. The selection of 500 patients was based on a post hoc power analysis, which indicated that this sample size was sufficient to detect statistically significant differences in morphometric measurements with ≥80% power. The sample also ensured the adequate representation of variability in anatomy across genders and age groups. While a priori power analysis was not performed, the inclusion of 500 participants allowed for the comprehensive analysis of gender-based differences and bilateral symmetry in the morphometric parameters. Although poor-quality CT scans were excluded, the potential effects of imaging resolution on the consistency of 3D reconstructions were addressed by standardizing the imaging protocols. Scans with slice thickness exceeding 1 mm or with motion artifacts were excluded to minimize variability. The limitations of CT resolution and their implications on the accuracy of 3D models were acknowledged as a factor warranting further investigation in future studies. These measures collectively ensured the quality and reliability of the data used for analysis.

### 2.2. Morphometric Measurements

**Figure 1:**
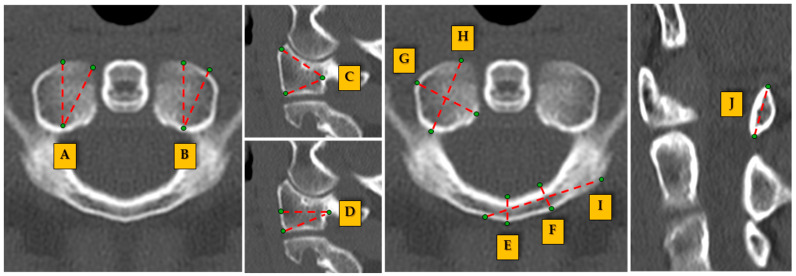
Visual representation of the measurement points and angles for the C1 vertebra.

Morphometric parameters were meticulously measured directly from the 3D models of the C1 and C2 vertebrae to capture comprehensive anatomical data crucial for surgical planning and navigation. The measurements were conducted as follows:

C1 measurements (Figure 1): (A) Angle of screw directed to maximal medial: the angle formed by a line directed medially through the lateral mass, representing the optimal trajectory for screw insertion in medial placement. (B) The angle of screw directed to the maximal lateral: the angle that extends laterally, ensuring safe and stable screw placement, while avoiding cortical breaches. (C) The angle of screw directed to maximal cranial: measured from the inferior aspect of the C1 lateral mass, this angle represents cranial screw trajectory. (D) The angle of screw directed to maximal caudal: the angle directed caudally from the C1 posterior arch, assessing the downward trajectory of the screws. (E) Posterior tubercle bicortical diameter: the bicortical diameter measured across the posterior tubercle, indicating the bone’s capacity to accommodate bicortical screw placement. (F) Bicortical diameter: the total diameter across the C1 lateral mass, aiding in understanding the safe diameter for bicortical screw fixation. (G) Transverse width: measured from one lateral mass to the other, providing insight into the width of C1 and the space available for surgical access. (H) Anteroposterior (AP) dimension: the distance measured from the anterior to the posterior aspects of the C1 arch, relevant for ensuring safe anterior or posterior screw placement. (I) C1 lamina length: the length of the lamina spanning the posterior arch of C1, crucial for posterior surgical approaches. (J) C1 lamina height: the vertical height of the C1 lamina, which contributes to understanding the space available for instrumentation.

**Figure 2:**
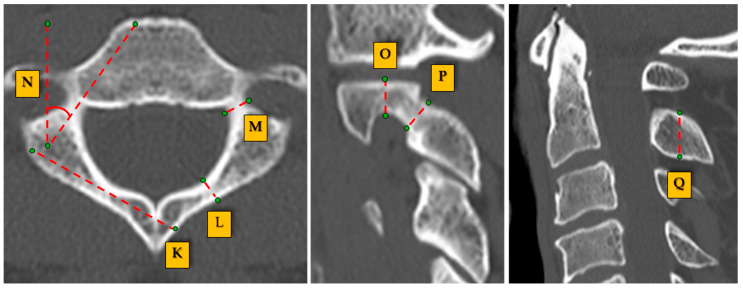
Detailed measurement points for the C2 vertebra.

C2 measurements (Figure 2): (K) C2 lamina length: the length of the C2 lamina from the base to the posterior edge, essential for posterior stabilization procedures. (L) C2 lamina bicortical diameter: the bicortical width measured across the lamina, aiding in determining the feasibility of bicortical screw placement. (M) Pedicle width: the transverse measurement across the C2 pedicle, crucial for assessing screw placement to avoid cortical breaches. (N) Pedicle transverse angle: the angle formed by a line drawn through the pedicle in a transverse plane, guiding the appropriate trajectory for pedicle screw insertion. (O) Internal height: the vertical distance from the internal cortical margin of the C2 lamina, indicative of the space available for instrumentation without cortical disruption. (P) Isthmus height: the height of the narrowest segment of the C2 lamina, serving as a limiting factor for screw placement and instrumentation. (Q) Height of the C2 lamina: the total vertical height measured from the upper to the lower cortical surfaces of the C2 lamina, influencing the choice of surgical tools and screw length.

The authors ensured precise measurements by using digital calipers within the InVesalius 3.1 software. Each parameter was measured three times independently by two observers, with the average value used for analysis. In cases of observer discrepancies, a senior spine surgeon reviewed the data to reach a consensus, ensuring accuracy and consistency.

### 2.3. Three-Dimensional Model Construction

**Figure 3:**
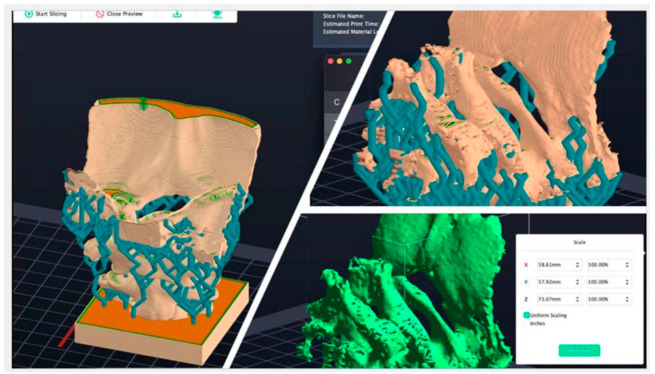
Three-dimensional printing reconstruction model of the cervical spine derived from CT imaging data.

CT scan data were processed using InVesalius 3.1 software (CTI, Campinas, São Paulo, Brazil) to generate high-resolution, three-dimensional digital models of the C1 and C2 vertebrae. The segmentation process involved isolating the vertebrae from the surrounding structures to achieve accurate anatomical representations. The models were subsequently refined and visualized using Meshmixer software (Version 3.5.0; Autodesk Inc., San Rafael, CA, USA), enabling the detailed analysis of their anatomical features. To validate the accuracy of the 3D models, the reconstructions were cross-referenced with established anatomical cadaveric data. The validation process involved comparing key morphometric parameters measured on 3D models with those from Raw file CT-Scan data to confirm their precision. Furthermore, two experienced observers independently reviewed the models, and discrepancies were resolved through consensus with a senior consultant. These steps ensured the reliability of the reconstructed anatomical data (Figure 3).

### 2.4. Data Collection and Observer Reliability

The measurements were independently conducted by two observers: an orthopedic surgeon with 3 years of experience and a senior spine specialist with 5 years of experience. To resolve any discrepancies between the observers, a senior consultant with over 15 years of experience reviewed the cases and provided a consensus. Interobserver reliability was assessed using the intraclass correlation coefficient (ICC), with values above 0.75, denoting excellent agreement. This reliability study was designed to satisfy the Quality Appraisal of Diagnostic Reliability (QAREL) checklist [11] as follows: Sample of subjects: the study analyzed cervical spine CT scans from 500 adult patients, ensuring a representative sample for the morphometric analysis. Sample of raters: the raters included an orthopedic surgeon with 3 years of experience and a senior spine specialist with 5 years of experience, while a senior consultant (10 years of experience) provided consensus. Blinding to other raters’ findings: observers were blinded to each other’s findings to reduce bias. Blinding to their own prior findings: observers were blinded to their prior results to minimize intra-observer bias. Blinding to the accepted reference standard: observers did not have access to the reference standard findings during initial measurements. Blinding to clinical information: clinical data unrelated to the specific measurements were concealed from the raters. Blinding to nonclinical cues: nonclinical cues, such as demographic information, were excluded from the measurement process. Order of examination: the examination order was randomized to prevent any potential order effects. Time interval between measures: the measurements were repeated after an appropriate time interval to reduce memory bias, while ensuring consistency. Correct test application and interpretation: all measurements adhered to a predefined, standardized protocol to ensure consistent application and interpretation. Appropriate statistical measures of agreement: Reliability was quantified using ICC values, with thresholds for excellent agreement (>0.75) consistent with established guidelines. This comprehensive approach minimizes bias, ensures high-quality data, and enhances the reliability of the study’s findings.

### 2.5. Statistical Analysis

Statistical analyses were performed using SPSS software (Version 29; IBM Corp., Armonk, NY, USA). Morphometric data were summarized as mean ± standard deviation (SD). Comparisons between male and female participants and assessments of bilateral symmetry were conducted using the Student’s *t*-test. Statistical significance was set at *p* < 0.05. A post hoc power analysis was conducted to evaluate the adequacy of the sample size in detecting statistically significant differences in morphometric measurements. Although a priori power analysis was not performed, the post hoc analysis provided an estimation of the achieved statistical power based on the observed effect sizes and the sample size of 500 participants (250 males and 250 females). The results indicated that the sample size was sufficient to achieve a statistical power of ≥80% for most evaluated parameters, thereby supporting the reliability of the study’s findings.

### 2.6. Ethical Approval

This study was approved by the institutional review board of Phayao University Hospital (IRB Number: HREC-UP-HSST 1.1/009/67) on 20 November 2023. Patient data were anonymized to protect confidentiality, and the need for informed consent was waived due to the retrospective nature of the study.

## 3. Results

### 3.1. Demographic Summary

The study analyzed a total of 500 adult patients, evenly divided between 250 males and 250 females, with a mean age of 45.8 ± 12.3 years (range: 18–75 years). All participants met the inclusion criteria, which required the absence of prior cervical spine surgery, significant spinal deformities, or conditions that could compromise morphometric measurements, such as severe osteoporosis or congenital abnormalities. Participants were selected from a single-center hospital database, ensuring uniformity in imaging protocols and data acquisition. The balanced gender distribution and wide age range allowed for a comprehensive analysis of gender-based and age-related variations in the morphometric parameters. This demographic diversity underscores the generalizability of the findings to a broad adult population. Furthermore, the selection process ensured the inclusion of high-quality CT scans, free from motion artifacts or imaging inconsistencies, thereby enhancing the reliability and accuracy of the subsequent 3D reconstructions.

### 3.2. Morphometric Characteristics of C1

Table 1 presents the detailed morphometric data of the C1 vertebra, comparing male and female participants. Significant gender-based differences were observed in several measurements. For instance, males had a greater transverse width (13.00 ± 1.45 mm) compared to females (12.70 ± 1.35 mm, *p* < 0.001), and the C1 lamina length was also longer in males (22.00 ± 1.75 mm) than in females (21.60 ± 1.65 mm, *p* = 0.033). Similarly, the C1 lamina height was slightly higher in males (7.85 ± 1.40 mm) compared to females (7.70 ± 1.30 mm, *p* = 0.006). Most measurements demonstrate consistent bilateral symmetry, as reflected in the comparison of the right and left sides. This uniformity supports the anatomical reliability of C1 as a reference for surgical planning. For example, the posterior tubercle bicortical diameter showed no significant differences between sides, ensuring predictable landmarks for bicortical screw placement.

### 3.3. Morphometric Characteristics of C2

Table 2 summarizes the morphometric data for the C2 vertebra, highlighting significant gender-based differences in key measurements. For example, males exhibited a greater pedicle width (4.85 ± 0.90 mm) compared to females (4.60 ± 0.85 mm, *p* = 0.033), and the height of the C2 lamina was higher in males (12.90 ± 1.40 mm) than in females (12.40 ± 1.25 mm, *p* < 0.001). Additionally, the internal height of the C2 lamina was larger in males (4.40 ± 0.80 mm) than in females (4.15 ± 0.75 mm, *p* = 0.001). Bilateral symmetry was observed in most parameters, indicating consistent anatomical landmarks across both sides. For instance, the pedicle transverse angle and the C2 lamina length showed no significant differences between the right and left sides, confirming the anatomical reliability of these features for surgical navigation.

### 3.4. Summary of Gender-Based Comparisons

Table 3 and Table 4 present a detailed comparison of morphometric measurements for the C1 and C2 vertebrae between male and female participants. Significant gender-specific differences were identified, particularly in parameters, such as pedicle width and the height of the C2 lamina. These differences highlight the importance of incorporating gender-specific anatomical considerations into surgical planning to optimize precision and safety. The morphometric analysis of 3D models provided valuable anatomical insights, demonstrating the utility of these models in preoperative planning. Additionally, the results revealed consistent bilateral symmetry in most measurements across genders. This symmetry underscores the reliability of the 3D models as dependable references for surgical navigation, enabling surgeons to use predictable anatomical landmarks and reducing variability during procedures. These findings emphasize the potential of 3D morphometric data in improving surgical outcomes and fostering patient-specific approaches.

## 4. Discussion

This study conducted a comprehensive morphometric analysis of the C1 and C2 vertebrae using high-resolution 3D models derived from CT scans, with a focus on gender-based differences and bilateral symmetry. The findings provide valuable insights with significant implications for surgical planning and clinical practice in cervical spine procedures. Notably, the results revealed significant gender-specific differences in key morphometric parameters, such as pedicle width and C2 lamina height. These variations are clinically important, as they influence the choice of surgical techniques and instrumentation. For example, the narrower pedicle width observed in female patients necessitates careful screw placement to avoid cortical breaches, while the height of the C2 lamina determines the appropriate screw length for stabilization procedures. These gender-specific differences underscore the importance of adopting personalized surgical approaches to enhance precision and improve patient safety in cervical spine surgery.

**Figure 4:**
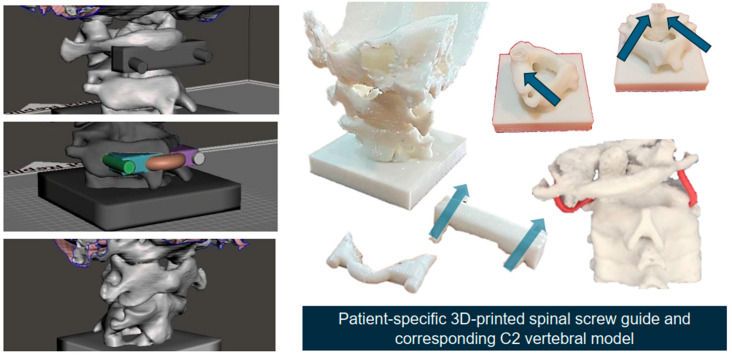
The 3D-printed models enhance the surgeons’ ability to visualize and assess anatomical variations, practice surgical techniques, and plan patient-specific procedures, contributing to improved surgical precision and patient outcomes. The patient-specific, 3D-printed spinal screw guides and corresponding C2 vertebral models are derived from CT imaging data. It highlights advancements in personalized spine surgery planning and execution. The dark blue arrows indicate the trajectory of the screws, while the light blue arrows represent the direction of the screw guides.

The observed consistency in bilateral symmetry across most morphometric measurements highlights the reliability of 3D models for preoperative planning. This aligns with prior research emphasizing the critical role of precise anatomical representations in improving surgical outcomes [12,13,14,15]. By offering detailed visualizations, the 3D modeling approach equips surgeons with a comprehensive understanding of complex anatomical structures, facilitating navigation and reducing potential risks during cervical spine surgery [9,16]. Figure 4 showcases 3D-printed models of the cervical spine, illustrating the practical application of high-resolution digital models in preoperative planning. These tangible models enable surgeons to visualize anatomical variations more clearly, practice complex procedures, and develop patient-specific surgical strategies. By integrating such models into clinical practice, surgical teams can enhance their understanding of challenging anatomical features, leading to improved precision during operations.

The findings of this study are consistent with and build upon prior research, offering significant advancements in understanding vertebral morphology. While earlier studies identified gender differences in vertebral structure, this study provides a more comprehensive analysis using high-resolution 3D reconstructions, which enhance accuracy and visualization compared to traditional two-dimensional imaging methods [9,14,16]. This detailed approach underscores the clinical relevance of morphometric variations in surgical decision making, offering surgeons precise data to inform their techniques. Accurate morphometric knowledge of the C1 and C2 vertebrae is crucial for the safe and effective placement of screws and other surgical instruments [17,18]. The data generated in this study enable surgeons to anticipate anatomical challenges and adjust their surgical strategies accordingly [19]. For instance, insights into average screw trajectory angles and the dimensions of pedicles and laminae are invaluable for optimizing screw placement and stabilization techniques, especially in complex surgical cases where precision is critical [14,20]. The study also highlights the importance of incorporating gender-specific considerations into surgical planning. Tailored approaches that address these anatomical differences can improve surgical outcomes and minimize complications. The findings advocate for the integration of 3D digital modeling as a standard tool in preoperative planning for cervical spine surgeries, emphasizing its potential to enhance precision and patient safety.

While this study provides significant insights and advancements over prior research, it has limitations. The retrospective nature of the study may introduce selection bias, and the sample was restricted to patients who met specific inclusion criteria, potentially limiting the generalizability of the findings to broader populations. Although the study references previous work, it primarily highlights its contributions without a direct comparative analysis. This study advances the field by offering precise, patient-specific 3D morphometric data, emphasizing gender-based differences and bilateral symmetry, which surpasses the scope of prior research relying on 2D imaging or limited parameters.

Future research should address these limitations by including larger, more diverse populations to enhance the applicability of findings. Additionally, exploring the use of 3D modeling in dynamic assessments could provide insights into how morphometric variations impact spinal biomechanics under various conditions. Another essential direction is investigating how these morphometric findings influence intraoperative outcomes and long-term recovery. Furthermore, integrating artificial intelligence and machine learning algorithms could significantly enhance the precision of 3D modeling, enabling automated morphometric analysis and supporting real-time surgical navigation. These advancements would address practical challenges, such as standardizing imaging protocols, and refine the application of 3D modeling in cervical spine surgery, ultimately improving surgical precision and patient outcomes.

## 5. Conclusions

This study provides detailed morphometric data on the C1 and C2 vertebrae using high-resolution 3D models derived from CT scans, revealing significant gender differences in key parameters, such as pedicle width and C2 lamina height. These findings emphasize the importance of gender-specific and patient-tailored approaches in cervical spine surgery to enhance surgical precision and reduce risks. The consistent bilateral symmetry observed across most measurements supports the reliability of using 3D models for preoperative planning. Despite the retrospective design and limited generalizability, this study underscores the potential of 3D modelling to improve surgical outcomes and encourages future research, incorporating larger, more diverse populations and advanced technologies, like artificial intelligence, for dynamic morphometric analysis and real-time surgical assistance.

## Figures and Tables

**Table 1:** Morphometric characteristics of C1.

Measurement (mm)	Total Mean ± SD	Male Mean ± SD	Female Mean ± SD	*p*-Value (Right: Male vs. Female)	*p*-Value (Left: Male vs. Female)
(A) Angle of screw directed to maximal medial	24.26 ± 2.92	24.50 ± 3.10	24.00 ± 2.70	0.873	0.062
(B) Angle of screw directed to maximal lateral	33.99 ± 7.69	34.20 ± 7.90	33.80 ± 7.50	0.809	0.852
(C) Angle of screw directed to maximal cranial	52.32 ± 4.12	52.10 ± 4.00	52.60 ± 4.20	0.339	0.170
(D) Angle of screw directed to maximal caudal	19.23 ± 1.70	19.50 ± 1.80	18.90 ± 1.60	0.872	0.845
(E) Posterior tubercle bicortical diameter	6.93 ± 1.84	7.00 ± 1.85	6.85 ± 1.82	0.014	0.011
(F) Bicortical diameter	5.63 ± 0.94	5.70 ± 0.95	5.55 ± 0.90	0.034	0.044
(G) Transverse width	12.85 ± 1.41	13.00 ± 1.45	12.70 ± 1.35	0.000	0.000
(H) AP dimension	18.57 ± 1.71	18.80 ± 1.75	18.35 ± 1.65	0.583	0.047
(I) C1 lamina length	21.79 ± 1.69	22.00 ± 1.75	21.60 ± 1.65	0.033	0.015
(J) C1 lamina height	7.78 ± 1.35	7.85 ± 1.40	7.70 ± 1.30	0.006	0.009

**Table 2:** Morphometric characteristics of C2.

Measurement (mm)	Total Mean ± SD	Male Mean ± SD	Female Mean ± SD	*p*-Value (Right: Male vs. Female)	*p*-Value (Left: Male vs. Female)
(K) C2 lamina length	20.98 ± 1.62	21.20 ± 1.60	20.75 ± 1.65	0.472	0.136
(L) C2 lamina bicortical diameter	4.44 ± 0.96	4.55 ± 1.00	4.35 ± 0.90	0.237	0.713
(M) Pedicle width	4.73 ± 0.87	4.85 ± 0.90	4.60 ± 0.85	0.033	0.036
(N) Pedicle transverse angle	44.63 ± 2.89	44.90 ± 2.95	44.35 ± 2.80	0.496	0.861
(O) Internal height	4.28 ± 0.79	4.40 ± 0.80	4.15 ± 0.75	0.001	0.001
(P) Isthmus height	5.82 ± 1.01	5.90 ± 1.05	5.75 ± 0.95	0.019	0.004
(Q) Height of the C2 lamina	12.65 ± 1.33	12.90 ± 1.40	12.40 ± 1.25	0.000	0.000

**Table 3:** Comparison of morphometric measurements between male and female participants for C1.

Measurement (mm)	Total Mean ± SD	Male Mean ± SD	Female Mean ± SD	*p*-Value
(A) Angle of screw directed to maximal medial	24.26 ± 2.92	24.50 ± 3.10	24.00 ± 2.70	0.354
(B) Angle of screw directed to maximal lateral	33.99 ± 7.69	34.20 ± 7.90	33.80 ± 7.50	0.712
(C) Angle of screw directed to maximal cranial	52.32 ± 4.12	52.10 ± 4.00	52.60 ± 4.20	0.489
(D) Angle of screw directed to maximal caudal	19.23 ± 1.70	19.50 ± 1.80	18.90 ± 1.60	0.112
(E) Posterior tubercle bicortical diameter	6.93 ± 1.84	7.00 ± 1.85	6.85 ± 1.82	0.452
(F) Bicortical diameter	5.63 ± 0.94	5.70 ± 0.95	5.55 ± 0.90	0.134
(G) Transverse width	12.85 ± 1.41	13.00 ± 1.45	12.70 ± 1.35	0.029
(H) AP dimension	18.57 ± 1.71	18.80 ± 1.75	18.35 ± 1.65	0.048
(I) C1 lamina length	21.79 ± 1.69	22.00 ± 1.75	21.60 ± 1.65	0.215
(J) C1 lamina height	7.78 ± 1.35	7.85 ± 1.40	7.70 ± 1.30	0.284

**Table 4:** Comparison of morphometric measurements between male and female participants for C2.

Measurement (mm)	Total Mean ± SD	Male Mean ± SD	Female Mean ± SD	*p*-Value
(K) C2 lamina length	20.98 ± 1.62	21.20 ± 1.60	20.75 ± 1.65	0.094
(L) C2 lamina bicortical diameter	4.44 ± 0.96	4.55 ± 1.00	4.35 ± 0.90	0.138
(M) Pedicle width	4.73 ± 0.87	4.85 ± 0.90	4.60 ± 0.85	0.048
(N) Pedicle transverse angle	44.63 ± 2.89	44.90 ± 2.95	44.35 ± 2.80	0.212
(O) Internal height	4.28 ± 0.79	4.40 ± 0.80	4.15 ± 0.75	0.021
(P) Isthmus height	5.82 ± 1.01	5.90 ± 1.05	5.75 ± 0.95	0.184
(Q) Height of the C2 lamina	12.65 ± 1.33	12.90 ± 1.40	12.40 ± 1.25	0.033

## Data Availability

The data presented in this study are available on request from the corresponding author. The data are not publicly available due to patient health data.
